# Easy and fast PCR‐based protocol allows characterization of breakpoints resulting from *Alu*/*Alu*‐mediated genomic rearrangements

**DOI:** 10.1002/mgg3.1830

**Published:** 2021-10-01

**Authors:** Filip Majer, Jakub Sikora

**Affiliations:** ^1^ Research Unit for Rare Diseases Department of Paediatrics and Inherited Metabolic Disorders First Faculty of Medicine Charles University General University Hospital Praha 2 Czech Republic; ^2^ Institute of Pathology First Faculty of Medicine Charles University General University Hospital Praha 2 Czech Republic

## CONFLICT OF INTEREST

The authors declare that they have no conflict of interest.

## AUTHOR CONTRIBUTIONS

FM drafted the initial version of the manuscript. JS and FM co‐edited the final version of the manuscript. FM submitted the manuscript.

**FIGURE 1:**
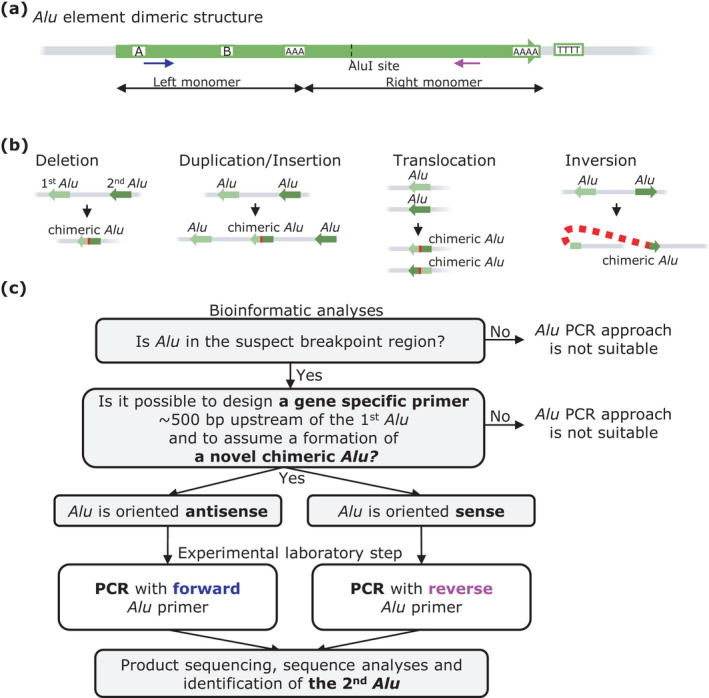
(a) A schematic of the key *Alu* sequence features. A and B box of polymerase III promoter, *AluI* cleavage site, two poly(A) tails and TTTT terminator sequence. Blue and purple arrows indicate positions and orientation of “*Alu* primers” used in the outlined PCR‐based method (Figure 2a‐c). (b) The main types of *Alu*‐mediated rearrangements. The interacting *Alus* are highlighted in light and dark green and are referred to as the 1^st^ and 2^nd^ *Alu*, respectively. The mutual orientation of the *Alus* determines the type of the genomic alteration. The recombination generates novel chimeric *Alu(s)*, the breakpoint junction is highlighted in red. (c) Graphical outline of the PCR‐based protocol for characterization of the AAMR breakpoints

Dear Editor,

Mobile repetitive sequences (retrotransposons) represent almost 44% of the human genome. *Alu* elements (*Alus*, Figure 1a) are the most abundant retrotransposons (Lander et al., 2001). The designation “*Alu* element” refers to the initial experiments showing that *AluI* restriction endonuclease cleaves these repetitive sequences (Houck et al., 1979). *Alus* are present exclusively in genomes of primates (Deininger et al., 1981) and evolved from the *7SL RNA* gene (Ullu, & Tschudi, 1984). This sequence accumulated changes over time and gave rise to *Alu* monomers. Fusion of these monomers later constituted the dimeric *Alu* that contains the free left *Alu* monomer (FLAM) and free right *Alu* monomer (FRAM; Figure 1a; Jurka, & Zuckerkandl, 1991). GC‐rich regions and a variable remnant of the poly A end are further features of *Alu* sequences (Figure 1a). Sequence differences categorize *Alus* into several subfamilies (*AluS*, *AluJ*, and *AluY*) and sub‐subfamilies (Batzer, & Deininger, 2002; Shen et al., 1991). Importantly, *Alus* contribute to genome evolution by retrotransposition and non‐allelic homologous recombination (Bailey et al., 2003).

*Alu*/*Alu*‐mediated rearrangements (AAMRs) are, however, also a very common source of pathogenic variants (mutations) resulting in human genetic diseases (Zhang et al., 2017). AAMRs cause copy number variations (CNVs) by introducing insertions and deletions. AAMRs may also result in translocations or inversions (Figure 1b). Recombination between two *Alus* often generates a new unique chimeric *Alu* (Gu et al., 2015; Konkel et al., 2015; Song et al., 2018) that is composed of portions of the original interacting *Alus* and is split by a microhomology region––the breakpoint (Song et al., 2018). Detailed characterization of these breakpoints is a critical prerequisite for correct interpretation of molecular genetic analyses in probands (and their families) with suspected AAMRs. Despite substantial advances in DNA sequencing techniques (e.g., exome/genome sequencing or long‐range sequencing), identification of AAMRs remains difficult.

**FIGURE 2:**
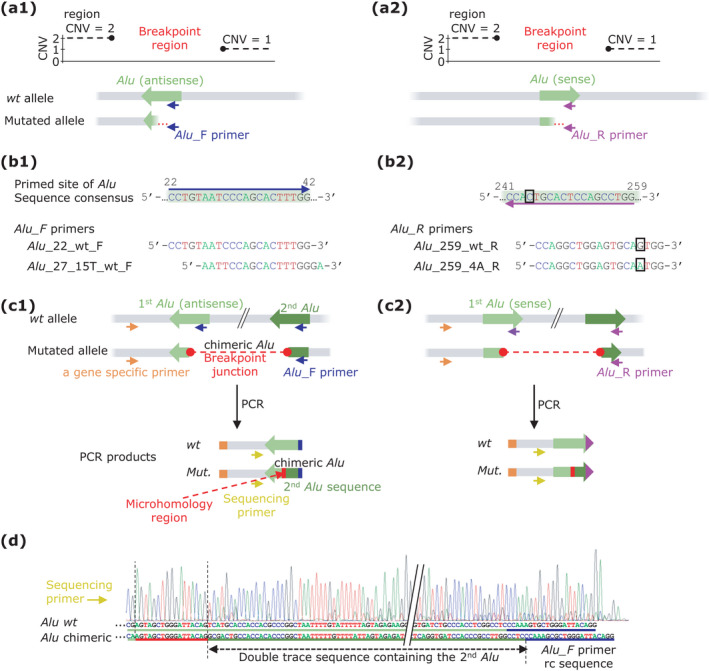
Characterization of the AAMR breakpoint in a model heterozygous genomic deletion is shown for two alternative orientations of the first interacting *Alu*. *Alu* elements are depicted in green. Depending on the orientation of *Alu* (antisense – a1 and sense – a2), the proximal or distal part of the *Alu* element is primed. *Alu* primers are marked as blue (*Alu* forward) and purple (*Alu* reverse) arrows. (b1 and b2) *Alu* primer binding site and its sequence consensus. *Alu* primer sequences to these sites are given. Primer Alu_259_4A_R for common variant (C to T at 244th base) in the region of 3′‐end of primer and an optional forward primer Alu_27_15T_wt_F are shown. (c1 and c2) A model AAMR mutation and illustration of the principle of the approach (explained in more detail in the main text). One PCR with one gene‐specific primer (orange) and one *Alu* primer yields a mixture of two PCR amplicons: a wild‐type and mutated allele. This product pair is then sequenced using a gene‐specific nested primer (lemon). (d) An example of electrophoretic trace and delineation of the mutated allele sequence from the wild‐type allele. The result is the identification of an admixed sequence of a novel chimeric *Alu* and thus the breakpoint site at the nucleotide level

To facilitate molecular genetic diagnostics, this Letter outlines a simple and effective PCR‐based approach that builds on sequence homology of *Alu* subfamilies. Specific primer design based on *Alu* consensus sequence (Price et al., 2004) allows characterization of AAMR‐induced breakpoints at the nucleotide level. We provide a general description of the protocol (Figure 1c) using characterization of a heterozygous genomic deletion as an example (Figure 2). The methodology can, however, be modified for other AAMR types.

First, the breakpoint region is roughly located by finding two neighboring genomic regions that differ in their relative copy number (Figure 2). Selection of the method (or combination of methods) is based on various aspects such as specificity of clinical findings in the tested individual(s), number of tested samples, availability of the necessary laboratory equipment (including post‐analytical data processing), or economic costs. While frequently used for CNV detection, next generation sequencing protocols remain compromised by a number of pitfalls (e.g., insufficient read length, proper read mapping, low or inhomogeneous coverage). We have previously used a combination of qPCR‐based DNA copy number analyses, whole exome sequencing with normalized coverage and mRNA analyses (Jedličková et al., 2020; Majer et al., 2020). Once estimated, we suggest performing bioinformatic inspection of the reference sequence for the presence of any *Alu(s)* in the suspected breakpoint region. If *Alu(s)* is/are present, AAMR should be suspected and formation of a novel chimeric *Alu* containing partial sequences of the two interacting *Alus* is likely. Attaining and sequencing a PCR product with the novel chimeric *Alu* allows more precise characterization of the breakpoint.

To generate such a PCR product, we suggest design of a region‐specific forward primer that aligns close (optimally <500 bp) to the suspected (first) *Alu*. The second PCR primer must be located downstream of the expected breakpoint. Our protocol presumes that the sequence of the novel chimeric *Alu* from the unknown (second) *Alu* contains the conserved region of the *Alu* family. A universal primer aligning to a highly conserved region of *Alu* element consensus (~300 bp long; Price et al., 2004) can, therefore, be effectively used to amplify the breakpoint junction (Figures 1c and 2).

Of note, candidate *Alu* elements can be oriented sense or antisense. Provided the first *Alu* is oriented *antisense*, the proximal part of the *Alu* consensus is primed with a universal *Alu* forward primer (Figure 2a1). However, when the first *Alu* is *sense* oriented, the distal part of the *Alu* consensus is primed with a universal *Alu* reverse primer (Figure 2a2). Design of these universal primers is complicated by the dimeric nature of *Alu* elements (Figure 1a) and the presence of GC‐rich regions and poly A sequences (Figure 1a). To allow easy implementation in the laboratory, Figure 2b1,b2 provide the sequences of universal *Alu* primers that we successfully tested. Numbering in the primer names is based on *Alu* consensus sequence of Price et al. (2004). There is a common variant in the *Alu* consensus sequence at the 244^th^ base (C to T, boxed in Figure 2b2) of the binding site of the Alu_259_wt_Reverse primer. We therefore present a complementary primer (Alu_259_4A_Reverse) to amplify chimeric *Alus* with this common variant. Similarly, we list a second primer Alu_27_15T_wt_Forward that can be used to increase the likelihood of successful amplification of sequences variable in the last bases of 3’‐end of the Alu_22_wt_Forward primer (Figure 2a2). The outlined approach can be easily modified to delineate the breakpoint junction from both sides (Jedličková et al., 2020; Majer et al., 2020).

Chemical composition of the PCR reaction(s) that use the region‐specific primer and one of the universal consensus *Alu* primers were standard (75 mM Tris‐HCl pH 8.8, 0.01% Tween 20, 2.5 mM MgCl_2_, 200 μM each dNTP, and Taq DNA polymerase 25 units/ml) and supplemented with (NH_4_)_2_SO_4_ (~20 mM). We also advise limiting the duration of the extension step to no longer than 30 seconds to prevent formation of PCR products originating from neighboring *Alus* by *Alu* universal primer (a phenomenon observed in genomic fingerprinting PCR; Nelson et al., 1989).

In the optimal scenario, products of the PCR reaction(s) contain two amplicons: one of wild‐type allele and one containing the sequence of the breakpoint junction of the presumed chimeric *Alu* (Figure 2c1,c2). The reaction products are further sequenced. Sequence addition of the chimeric *Alu* to the wild‐type *Alu* can be resolved (Figure 2d). The portion of the sequence originating from the second *Alu* can be used for bioinformatic identification. In case the sequence of the second *Alu* is too short (e.g., as an effect of close localization of the universal *Alu* primer binding site to the breakpoint junction) it can still be visualized as a motif in an interface like The Integrative Genomics Viewer (Robinson et al., 2011) which helps to identify it. Other pitfalls to this approach that were encountered include: (i) the breakpoint might be outside of *Alu* or too close to the binding site of the universal *Alu* primer(s), (ii) interacting *Alus* are complex or atypical, and (iii) the novel chimeric *Alu* contains rare nucleotide variants in the primed site(s). Despite these limitations, the key advantages of our one‐step PCR approach are simplicity and speed. The necessary technical equipment is widely available in molecular genetic diagnostic laboratories.

In summary, we present a PCR‐based protocol that allows direct and fast mapping of even very complex *Alu*/*Alu*‐mediated rearrangements. We believe this method may substantially facilitate nucleotide level molecular diagnostics as well as family screening/counselling.

## Data Availability

Data sharing not applicable – no new data generated.
